# Identification of a BAZ2A-Bromodomain Hit Compound by Fragment Growing

**DOI:** 10.1021/acsmedchemlett.2c00173

**Published:** 2022-08-03

**Authors:** Andrea Dalle Vedove, Giulia Cazzanelli, Laurent Batiste, Jean-Rémy Marchand, Dimitrios Spiliotopoulos, Jessica Corsi, Vito Giuseppe D’Agostino, Amedeo Caflisch, Graziano Lolli

**Affiliations:** † Department of Cellular, Computational and Integrative Biology - CIBIO, University of Trento, via Sommarive 9, 38123 Povo - Trento, Italy; ‡ Department of Biochemistry, University of Zürich, Winterthurerstrasse 190, CH-8057 Zürich, Switzerland

**Keywords:** BAZ2A bromodomain, Prostate cancer, Fragment growing, X-ray crystallography, Molecular docking, Binding assays

## Abstract

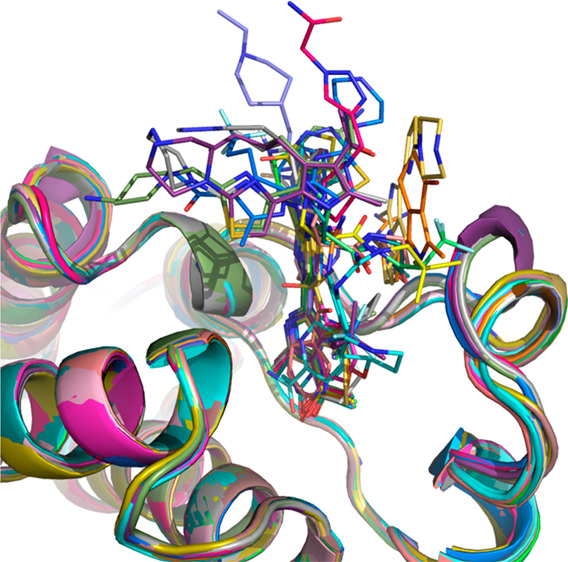

BAZ2A is an epigenetic regulator affecting transcription of ribosomal RNA. It is overexpressed in aggressive and recurrent prostate cancer, promoting cellular migration. Its bromodomain is characterized by a shallow and difficult-to-drug pocket. Here, we describe a structure-based fragment-growing campaign for the identification of ligands of the BAZ2A bromodomain. By combining docking, competition binding assays, and protein crystallography, we have extensively explored the interactions of the ligands with the rim of the binding pocket, and in particular ionic interactions with the side chain of Glu1820, which is unique to BAZ2A. We present 23 high-resolution crystal structures of the holo BAZ2A bromodomain and analyze common bromodomain/ligand motifs and favorable intraligand interactions. Binding of some of the compounds is enantiospecific, with affinity in the low micromolar range. The most potent ligand has an equilibrium dissociation constant of 7 μM and a good selectivity over the paralog BAZ2B bromodomain.

Bromodomains are epigenetic readers of acetylated lysines (Kac) in histones and other proteins. The human genome comprises 61 bromodomains in 46 proteins that also include additional DNA or protein-recognition/modifying domains (acetylases, PHD zinc fingers, helicases, and methyltransferases).^1^ Due to their involvement in the regulatory machinery of chromatin conformation and accessibility, bromodomains have been recognized as promising drug targets, especially in cancer and inflammatory diseases. Various small-molecule inhibitors have been developed, with some of them in clinical trials.^2^

BAZ2A (bromodomain adjacent to zinc finger domain protein 2A) is one of the nucleolar remodeling complexes mediating chromatin condensation and silencing of ribosomal DNA (rDNA).^3,4^ However, its role extends beyond the epigenetic modulation of rDNA transcription. In prostate cancer, BAZ2A is overexpressed and cooperates with EZH2 (enhancer of zeste homolog 2) in potentiating tumor cells’ migration and metastatic potential.^5,6^ BAZ2A has then been proposed as a prognostic marker for aggressive and recurrent prostate cancer as well as a promising therapeutic target. BAZ2A inhibition has been found to synergize with BET (bromodomain and extra-terminal domain) inhibitors in suppressing growth in TNBC (triple-negative breast cancer) cells.^7^ Very effective anti-proliferative activity was obtained by simultaneous inhibition of the BET bromodomains and the non-BET members BRD9 and BAZ2A.

BAZ2A, however, features a shallow pocket, making it one of the least druggable bromodomains.^8^ Only two chemical probes have been developed so far, namely GSK2801 and BAZ2-ICR, with affinities in the very high nanomolar range.^9,10^ A rationale for improving BAZ2A inhibitors is to look for additional interactions with residues located at the tip of its binding pocket.^11^

Here we present the optimization of a series of ligands of the BAZ2A bromodomain by structure-based fragment growing.

An initial set of about 50 000 molecules was selected in parallel using two different computational methods: a protein-based search and a ligand-based procedure. The first method consisted of a Pharmit search^12^ starting from a parent fragment identified in a previous screening campaign (compound **1** in ref (11)) for the pharmacophoric definition. The ligand-based procedure consisted of a selection of the ZINC15 library^13^ limited to molecules with up to three rotatable bonds. In both cases, only compounds bearing a positive charge were kept, with the aim of reaching BAZ2A Glu1820 at the rim of the pocket as a putative determinant for increased potency and selectivity (Figure S1, structural alignment of representative bromodomains). The resulting 50 000 molecules were screened by automatic docking (see the Supporting Information for details). The docked poses were ranked by a force-field-based energy function with approximation of desolvation effects in the continuum-dielectric representation.^14,15^ A total of 80 molecules were selected, for which commercial availability was checked, and 36 were obtained (Table S1).

The 36 selected molecules were tested by AlphaScreen at single doses in duplicate. Six molecules (compounds **8**, **9**, **11**, **14**, **18**, and **25**) decreased binding of BAZ2A bromodomain to the acetylated peptide by at least 50% when tested at 500 μM. The IC_50_ was evaluated for those compounds, resulting in binding inhibition in the high micromolar range, except for compound **25**, which performed significantly better than the others (Figure S2 and Table 1).

**Table 1:**
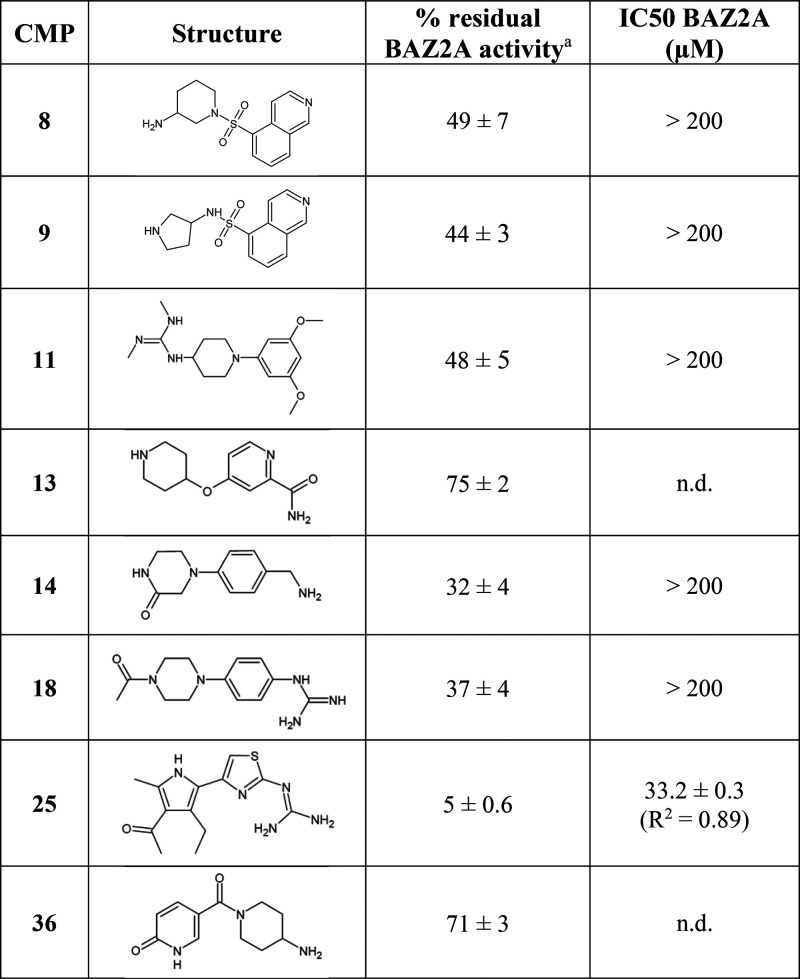
BAZ2A Binding Fragments Identified by Docking and Validated by Competitive Binding Assay or X-ray Crystallography

a The single-dose experiment was carried out at a compound concentration of 500 μM.

All 36 fragments were also screened in co-crystallization experiments with the BAZ2A bromodomain. For about half of the compounds, crystals did not grow or only diffracted to very poor resolution, including the active fragments **8**, **11**, and **14**, suggesting an interference with the crystallization process. Various BAZ2A apo crystals were obtained in co-crystallization with compounds showing <20% inhibition at 500 μM, confirming they are not BAZ2A binders. Co-crystals were obtained with fragments **9**, **13**, **18**, **25**, and **36**.

All compounds bind in the Kac pocket with their respective headgroups sandwiched between Val1822 and Val1879, anchoring through a hydrogen bond to side chains of Asn1873 or Tyr1830 (via the conserved W1 water molecule) or both (Figure 1a).

**Figure 1:**
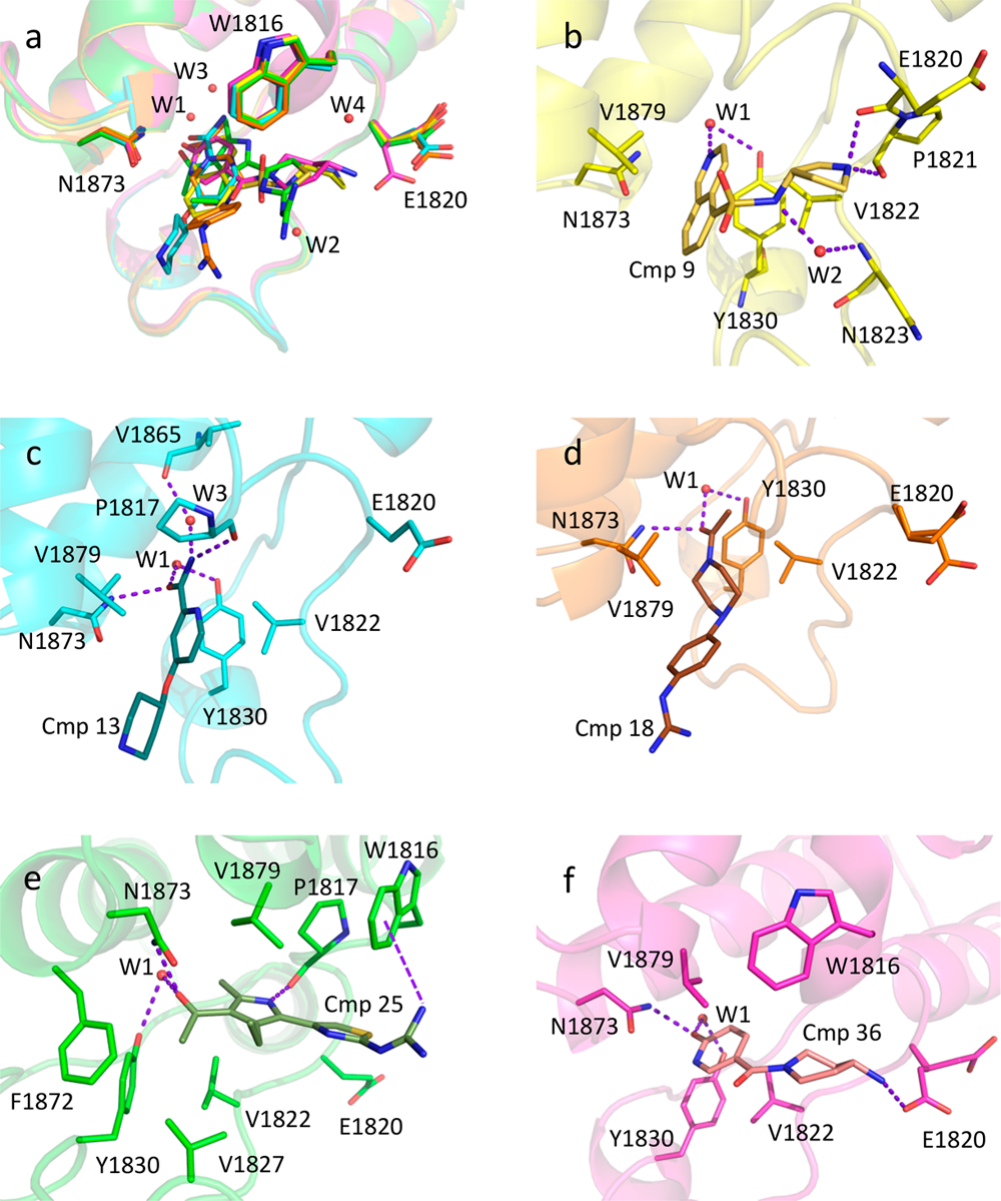
Binding mode of fragments to the BAZ2A bromodomain. (a) Headgroups of the different fragments are involved in similar contacts with the protein matrix. (b–f) Binding poses of compounds **9** (b), **13** (c), **18** (d), **25** (e), and **36** (f).

In compound **9**, the only direct H-bonds with the protein matrix are between the pyrrolidine nitrogen and both Glu1820 and Pro1821 main-chain oxygens, while the interactions of the isoquinoline nitrogen with Tyr1830 and of the sulfonamide nitrogen with Asn1823 main-chain nitrogen are mediated by W1 and W2 (Figure 1b). Despite the limited resolution, the electron density supports the largely preferential, if not exclusive, binding to BAZ2A of the single S enantiomer.

The binding mode of compound **13** is unexpected (Figure 1c). It runs parallel to the end of the αB helix, similarly to the Kac residue of acetylated peptides, instead of facing the WPF shelf of the ZA loop. Consequently, the charged piperidine is not directed toward Glu1820 but rather exposed to the solvent. The amide of compound **13** does not reciprocally interact with the side chain of Asn1823, unusually placing the amide nitrogen in the small pocket reserved for the KAc methyl group in physiological ligands. While the amide oxygen interacts with both Asn1823 and Tyr1830 (W1-bridged), the amide nitrogen establishes H-bonds to main-chain oxygens of Pro1817 and Val1865, the latter mediated by a water molecule (W3).

Compound **18** binds with a similar orientation with respect to **13** (Figure 1d). The **18** acetyl group, almost superposed to the **13** amide, again contacts Asn1823 and Tyr1830 through its oxygen, while the methyl group is correctly positioned in the corresponding hydrophobic pocket. The charged guanidinium is exposed to the solvent far away from Glu1820.

In compound **25**, the acetyl-pyrrole headgroup shows extended interactions with the protein matrix (Figure 1e). The pyrrole nitrogen is in H-bond contact with the Pro1817 main-chain oxygen. The methyl group in position 2 nicely fits the corresponding KAc methyl cavity. The 3-acetyl substituent forms the usual H-bonds with Asn1873 and Tyr1830, while the methyl group is in hydrophobic contact with Val1827, Tyr1830, and Phe1872. The ethyl group in position 4 contributes through hydrophobic and van der Waals interactions with Val1822, Val1827, and Val1879. The thiazole-guanidine moiety is rotated by about 180° with respect to the expected pose. While the thiazole ring is in π–stacking interaction (T-shaped) with Trp1816 as expected, the guanidinium group is not directed toward Glu1820 but instead forms a π–cation interaction again with Trp1816, although with sub-optimal geometry.^16^ The above-reported interactions support the relevant binding affinity observed for compound **25**.

The pyridone oxygen of compound **36** consistently interacts with Asn1823 and Tyr1830 (Figure 1f). The amino-piperidine tail is in hydrophobic and van der Waals contact with Trp1816; its charged, terminal nitrogen finally contacts the Glu1820 side chain. Electron density indicates that, together with the predominant *pt* conformer (χ1 = 63°) forming the ionic interaction with compound **36**, Glu1820 also assumes the *mm* rotameric form (χ1 = −65°), exposing its carboxylate to the solvent. This suggests that contribution to the binding energy of the salt bridge with Glu1820 is limited, at least for this compound, while potentially relevant for selectivity against other bromodomains.

We then set out to optimize fragment **25**. A sub-structure search on the ZINC15 database of commercially available compounds was conducted using the 3-acetyl-2,4-dimethyl-5-thiazole-pyrrole structure as a query. Compounds **37**–**73** (Table S2), all bearing a substituent in position 2 of the thiazole ring, were selected and their activities tested on the BAZ2A bromodomain by Alphascreen in single doses of 500 μM (Figure S3).

Compounds **37**–**41**, all bearing an aromatic 6-membered ring conjugated to the thiazole ring, were poorly active. Compounds substituted with aromatic 5-membered rings displayed some activity, although not evenly (e.g., **69** vs **68**). Nonplanar 5- or 6-atom heterocycles are generally well accepted, either directly linked to the thiazole ring or with a bridging methyl group, as well as linear groups. Generally, positively charged substituents performed better than polar groups (e.g., **49** vs **51**), while hydrophobic derivatives were ineffective (e.g., compound **42**).

Compounds showing inhibition of the BAZ2A bromodomain binding to the acetylated peptide by at least 67% in single doses were further tested for dose–response, together with the minimal compound **43** as reference. This screen identified that compound **47** was as active as **25** (Table 2 and Figure S4). Binding affinity is influenced by the number and location of nitrogen atoms in the tail groups. This can be deduced when comparing compounds **47** (holding a piperazine ring) and **48**–**50** (4-, 3-, and 2-piperidine). The favorable contribution deriving from a positive charge is again evident when comparing **53** and **54** with **55**–**58** (charged vs uncharged pyrrolidine rings).

**Table 2:**
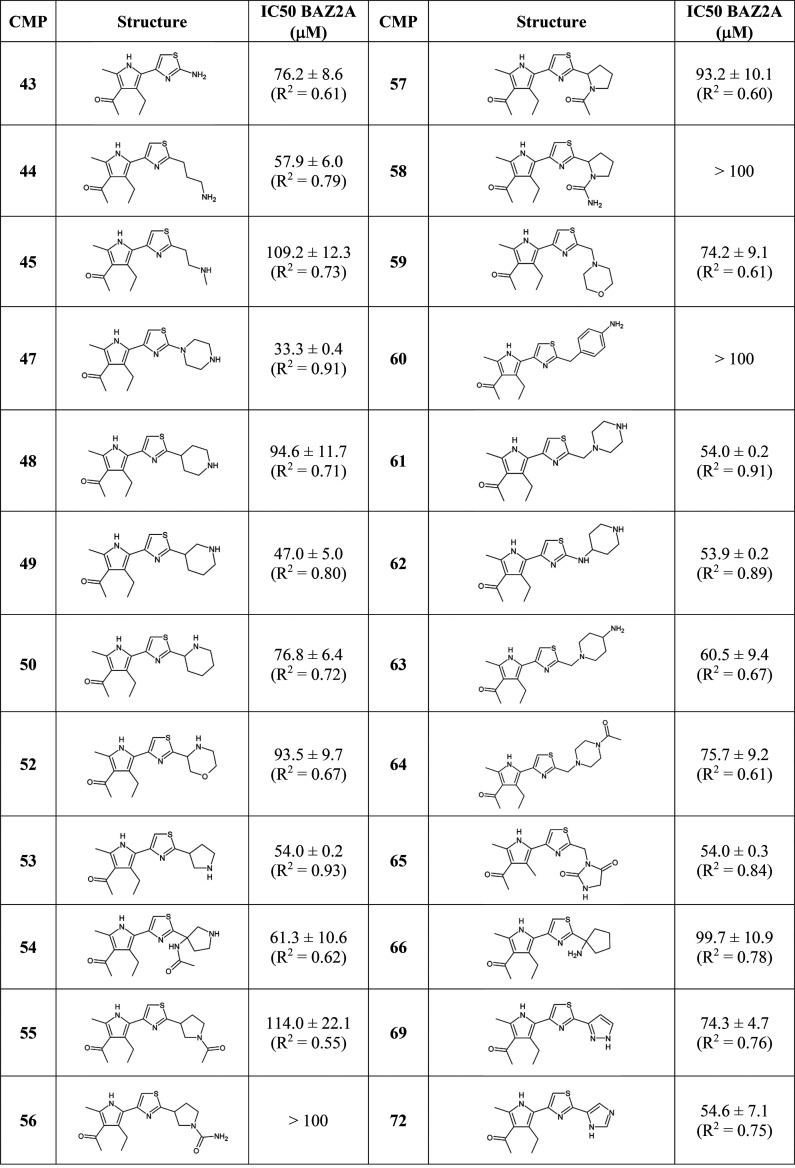
Activity of Conjugated Acetylpyrrole-Thiazole Compounds as BAZ2A Binders

We determined the crystallographic structures for six compounds in complex with BAZ2A, namely **44**, **45**, **47**, **61**, **63**, and **65**. The most striking difference between these compounds and **25** is the 180° rotation of the thiazole ring with the subsequent different orientations assumed by the tails departing from it (Figure 2a).

**Figure 2:**
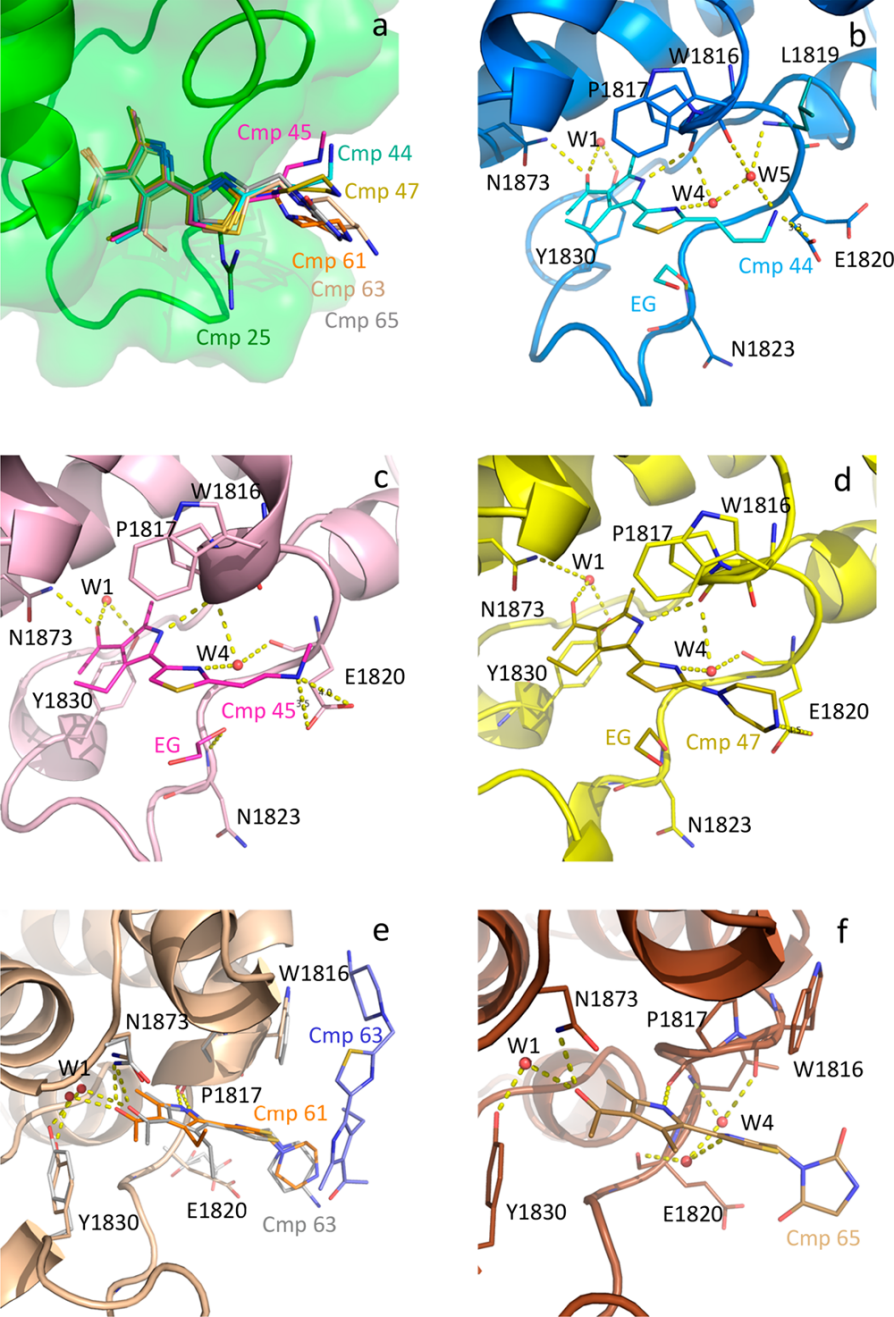
Binding mode of acetylpyrroles to the BAZ2A bromodomain. (a) Compounds are almost identically placed in their headgroup region but differ in the orientation of their tail moieties. (b–f) Binding poses and interactions for compounds **44** (b), **45** (c), **47** (d), **61** (e), **63** (f), and **65** (g). In panels b–d, the ethylene glycol molecule is indicated with EG. In panel e, compound **61** is in orange, the two copies of compound **63** are in gray and violet, and relevant amino acids are in wheat (in the complex with **61**) and white (in the complex with **63**).

The 3-aminopropyl substituent in compound **44** performs better than the *N*-methyl-2-aminoethyl group of **45**. Binding poses of compounds **44** and **45** are similar: all interactions of the acetyl-pyrrole headgroup are conserved with respect to **25**, but the charged tails do not point toward Trp1816, but instead approach Glu1820 (Figure 2b,c). However, as observed in compound **36**, Glu1820 oscillates between two conformers, one in contact with the compound amino group and the other exposed to the solvent; this interaction seems to contribute only partially to the binding affinity. Instead, compound **44** is involved in a network of water-mediated interactions with the protein matrix (Figure 2b). The thiazole nitrogen forms a hydrogen bond with water W4, which tetrahedrally interacts with the main chain of Pro1817 and water molecules W5 and W6, in turn in contact with BAZ2A. Water W5 is tetrahedrally coordinated by the terminal amino group of **44**, the Trp1816 main-chain oxygen, the Leu1819 main-chain nitrogen, and W4. The interaction between the secondary amine and W5 is missing in compound **45**, justifying its lower affinity when compared to **44** (Figure 2c). Despite the observed interaction patterns, both compounds have poor affinity for BAZ2A, most probably due to the entropic penalty deriving from the freezing of the alkylamine tails and the surrounding water network.

Compound **47** binds similarly to **44** and **45**. Better affinity derives from increased rigidity and bulkiness, resulting in additional van der Waals contacts with Trp1816 (Figure 2d). Similarly, the extra rigidity of the **47** piperazine with respect to the piperidine rings of compounds **48**–**50**, where the thiazole-linked planar nitrogen is substituted with a tetrahedral carbon, may explain the observed preference for the piperazine ring. Notably, in the structures in complex with **44**, **45**, and **47**, an ethylene glycol molecule is sandwiched between the thiazole ring and the ZA loop (Figure 2b–d). It forms a hydrogen bond with the main-chain nitrogen of Asn1823, an important anchoring point already explored by the BAZ-ICR and GSK2801 chemical probes and other BAZ2A inhibitors,^9,10,14^ and a relevant indication for further optimization of this chemical class.

Slightly more protruding tails are present in compounds **59**–**65** (Figure 2a). Compounds **59**, **61**–**64**, and, more unexpectedly, compound **65** (imidazolidinedione tail) retained a good activity although lower than compound **47**. The **61** piperazine ring faces Glu1820 at a distance of 4 Å (Figure 2e). The more protruding amino-terminal of compound **63** interacts with Asp1798 of a symmetry-related chain. Given the very similar activity of the two compounds, it is reasonable to suppose that Asp1798 from the symmetric mate artifactually competes in the crystal with Glu1820 for the binding to **63**. Again, and in both cases, the contribution to the binding energy of the ionic interaction with Glu1820 appears limited. Interestingly, an additional copy of compound **63** is present at the interface between symmetry-related protein chains, with its 1-(2-thiazolylmethyl)-4-piperidinamine moiety stacking almost parallel to Trp1816 (Figure 2e). This additionally suggests a derivatization vector for the inhibitor series pointing away from the ZA loop, i.e., in the opposite direction as the one previously exploited by ethylene glycol.

In compound **65**, the imidazolidinedione is oriented perpendicularly to the thiazole ring, better complementing the bromodomain surface through increased contacts with Trp1816 (Figure 2f).

Overall, the fragment expansion campaign defined a greater convenience in optimizing the inhibitor tail for its interaction with Trp1816 rather than with the solvent-exposed and flexible Glu1820, also suggesting growing vectors for the subsequent hit identification round.

Compound **47** was chosen for further optimization due to its higher versatility for further derivatization. In position 3 of the pyrrole ring, the acetyl group was changed to an amide or an *N*-propyl-amide in compounds **74** and **75** (Table 3), respectively. This attempt to improve interactions with Phe1872-Asn1873 failed, and the two compounds lost binding efficiency (Table 3 and Figure S5).

**Table 3:**
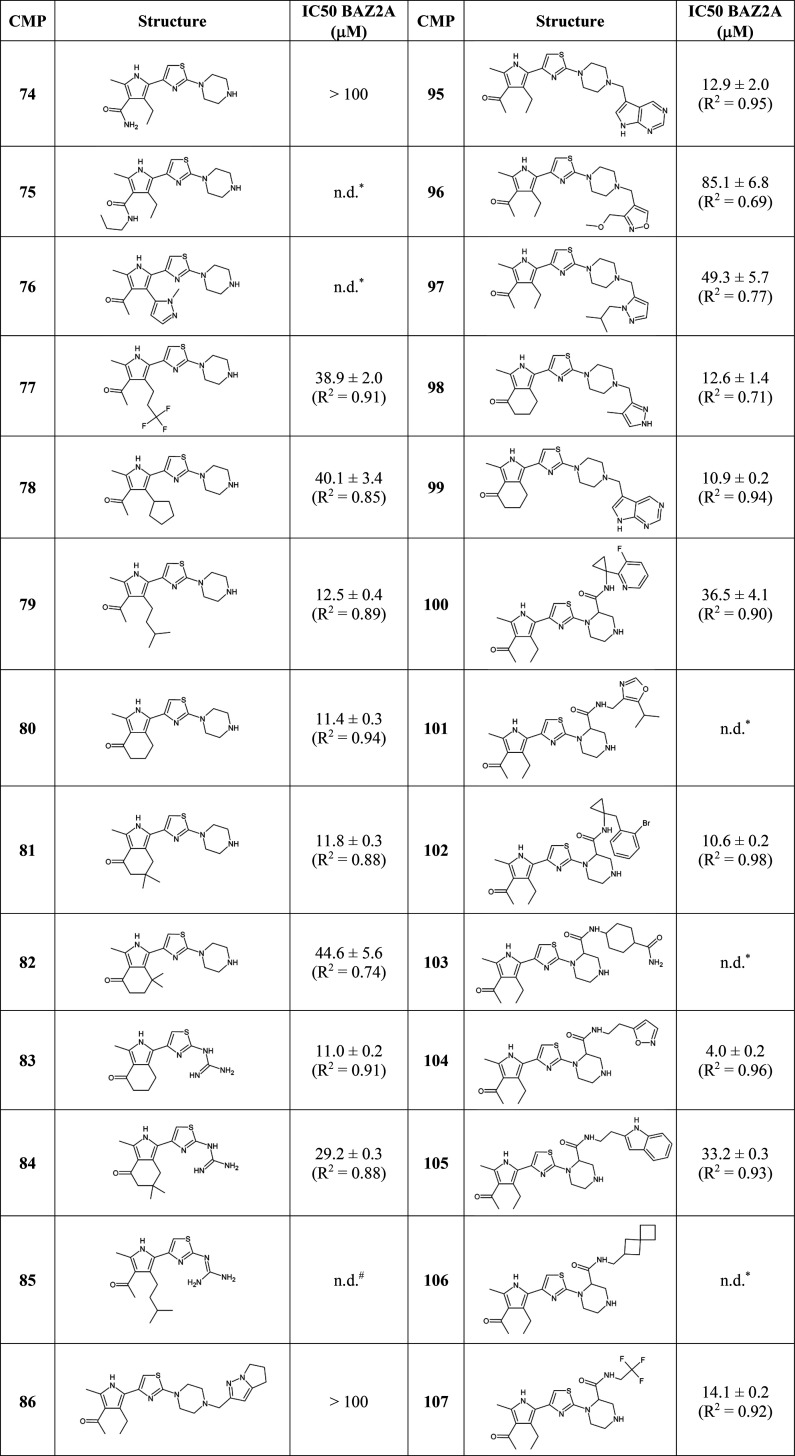

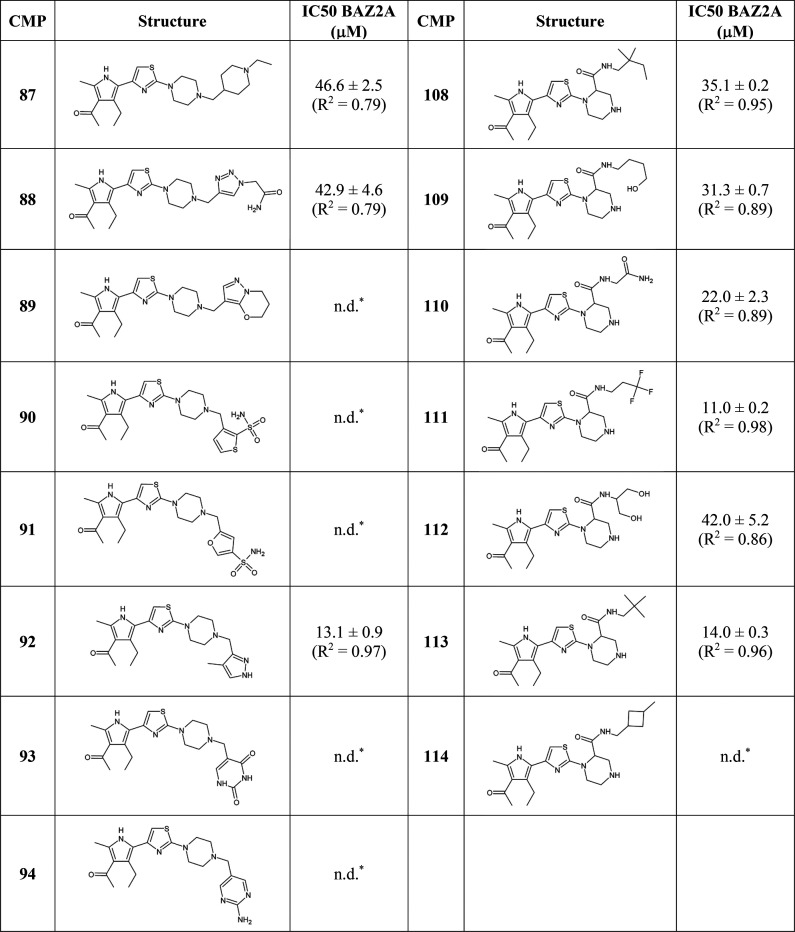
Compounds Screened in the Fragment-Growing Campaign^a^

a n.d. = not determined: *Residual activity >50% when tested in single dose at 100 μM. ^#^Compound rapidly crystallizing in the protein and assay buffers.

We then concentrated on three derivatization points in compound **47**: position 4 of the acetyl-pyrrole ring and positions 2 and 4 of the piperazine ring retrieving 88, 1156 (tertiary amine), and 26 295 (amide) synthesizable analogs, respectively. Following docking, clustering, and evaluation of synthetic convenience, compounds **76**–**114** were selected (Table 3).

The ethyl group at position 4 of the pyrrole ring points toward the entrance of the Kac pocket in the path occupied by the aliphatic Kac portion in peptidic ligands. Its substitution with a planar diazole ring in compound **76** (Table 3) significantly reduced binding to the BAZ2A bromodomain. Derivatives **77** and **78**, bearing a trifluoropropyl and a cyclopentyl substituent, respectively, did not perform substantially better than the parent compound **47** (Table 3 and Figure S5). The IC_50_ values for the isoamyl derivative **79** and the bicyclic tetrahydroisoindol-4-one analogs **80** and **81** are 3 times lower than that measured for **47** (Table 3 and Figure S5).

Crystallographic structures of compounds **77**–**80** (Figure 3a) show almost superimposable poses for the five inhibitors. Notably, the 0.98 Å resolution for the crystal structure of the complex with compound **77** is the highest achieved so far for BAZ2A. The extra trifluoromethyl group in compound **77** only makes very limited additional van der Waals contacts with Val1827 compared to **47** (Figure 3b). Extensive contacts with Leu1826 and Val1827 are formed by the bulkier substituents of compounds **78**–**79** (Figure 3c,d). However, the cyclopentyl group in compound **78** is squeezed between the two above residues, Val1879 and the thiazole ring, with excessive crowding in this region. This is confirmed in compounds **80**–**84**, where the gain obtained in **80** and **83** through cyclization of the headgroup is not reinforced by the extra methyl groups in **81**, **82**, and **84**. Comparison of binding poses for compounds **80** and **83** confirms the different orientations of the piperazine and guanidinium tails (Figure 3a,e,f), as observed for **25** and **47** (Figure 2a).

**Figure 3:**
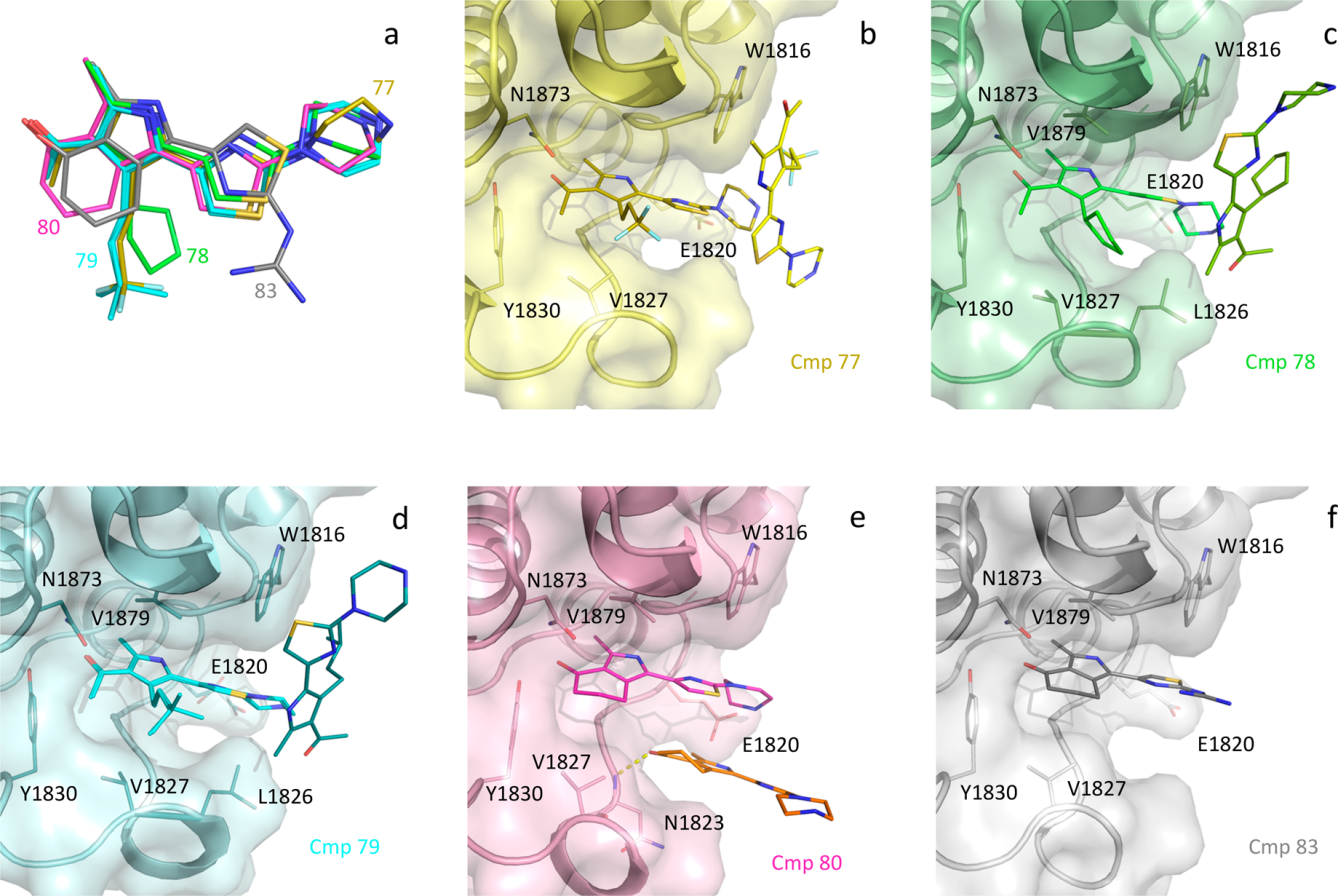
Binding poses of compounds **77**–**80** and **83** (a). A second copy of the inhibitor is present, stacking either to W1816 (compounds **77**, **78**, and **79**, panels b, c, and d, respectively) or to itself and hydrogen bonding to N1823 main-chain nitrogen (**80**, panel e). The tail of compound **83** is oriented differently with respect to the other compounds (panels a and f).

For compounds **86**–**99**, tails in position 4 of the piperazine ring were designed to stack with Trp1816 via π–π or cation−π interactions. None of them resulted in improved affinity with respect to compound **47** (Figure S5), suggesting that stacking interaction with the solvent-exposed Trp1816 does not significantly contribute to the binding energy. Indeed, in the BAZ2A-compound **98** co-crystal structure, the methyl-pyrazole tail contacts Trp1816 and Leu1819 as expected, but no significant improvement in affinity is appreciable (Figure 4a). Surprisingly, compound **88**, bearing a similar acetamide-triazole tail, assumes a different pose, with the thiazole ring rotated by 180° (as observed in **25**) and the subsequent acetamide-triazole tail fully solvent-exposed (Figure 4b). Similarly, the IC_50_ value for compound **87** is not lower than that for **47**; here, the piperazine ring is located as in the parent molecule **47** with its N4 atom at 3.9 Å from Glu1820 and also in hydrogen-bond contact with a water molecule bridging the inhibitor to Trp1816 carbonyl oxygen (Figure 4c). However, the extra ethyl-piperidinyl group protrudes linearly from the pocket and is exposed to the solvent without additional interaction with the protein matrix.

**Figure 4:**
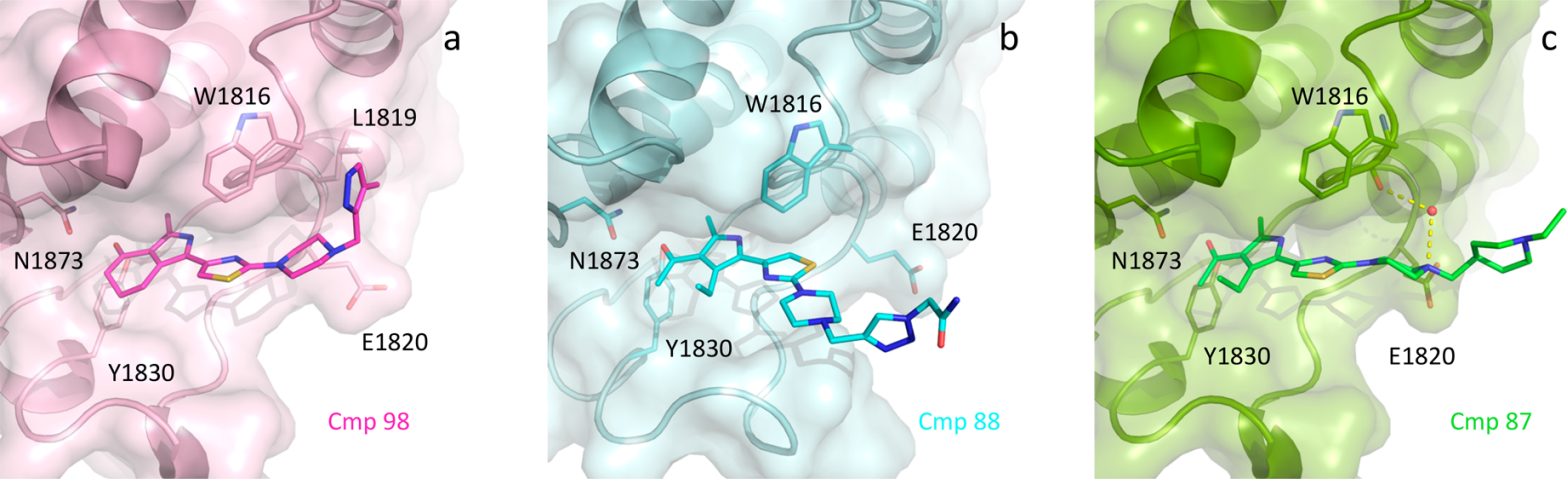
Binding poses of compounds **98**, **88**, and **87**. The tail of **98** is in contact with Trp1816 and Leu 1819 (a), while in compounds **88** and **87** (b, c) the tails are exposed to the solvent with minimal contacts with the protein matrix.

Derivatization in position 2 of the piperazine ring was more successful, yielding six compounds with improved affinity and, among these, compound **104** with an 8-fold increase in potency with respect to **47** (Table 3 and Figure S5). Notably, the piperazine C2 is a stereocenter in this class of compounds, and all of them were tested as racemic mixtures. We determined the crystallographic structures for compounds **104**, **109**, **111**, and **113**; in all of them, the thiazole ring reverses to the orientation observed in compound **25**, and only the R enantiomer binds in the BAZ2A pocket. Compounds **111** and **113** bear a hydrophobic tail, providing additional contacts with Leu1826 and Val1827 (Figure 5a). An ethylene glycol molecule, located as in the structures with compounds **44**, **45**, and **47**, also connects the amide nitrogen of **111** to the main-chain nitrogen of Asn1823. Compound **109** establishes the same H-bond with Asn1823 main chain through its terminal hydroxyl group (Figure 5b); however, an entropic penalty is conceivable considering the significant flexibility of the *n*-butanol tail. The higher affinity of **104** is evident: the ethyl-isoxazole tail turns back into the pocket, with its aromatic ring stacking almost parallel to the thiazole ring and establishing van der Waals interactions with Leu1826 and Val1827 and the hydrogen bond with Asn1823 main-chain nitrogen (Figure 5b). The important contribution of this H-bond to BAZ2A inhibitors has already been reported in both BAZ2 chemical probes GSK2801 and BAZ2-ICR and in a triazole-based BAZ2A hit compound. These last two also reproduce the four-layer sandwich observed in **104**, with the two aromatic rings squeezed between Trp1816 and Leu1826 (Figure 5c).^9,10,17^ The decreased ligand efficiency (LE) of **104** compared to the parent compounds **25** and **47** (0.24 vs 0.31 and 0.29 kcal/mol per heavy atom, respectively) is compensated however by a more favorable lipophilic ligand efficiency (LLE, 4.0 vs 2.4 and 1.9, respectively).

**Figure 5:**
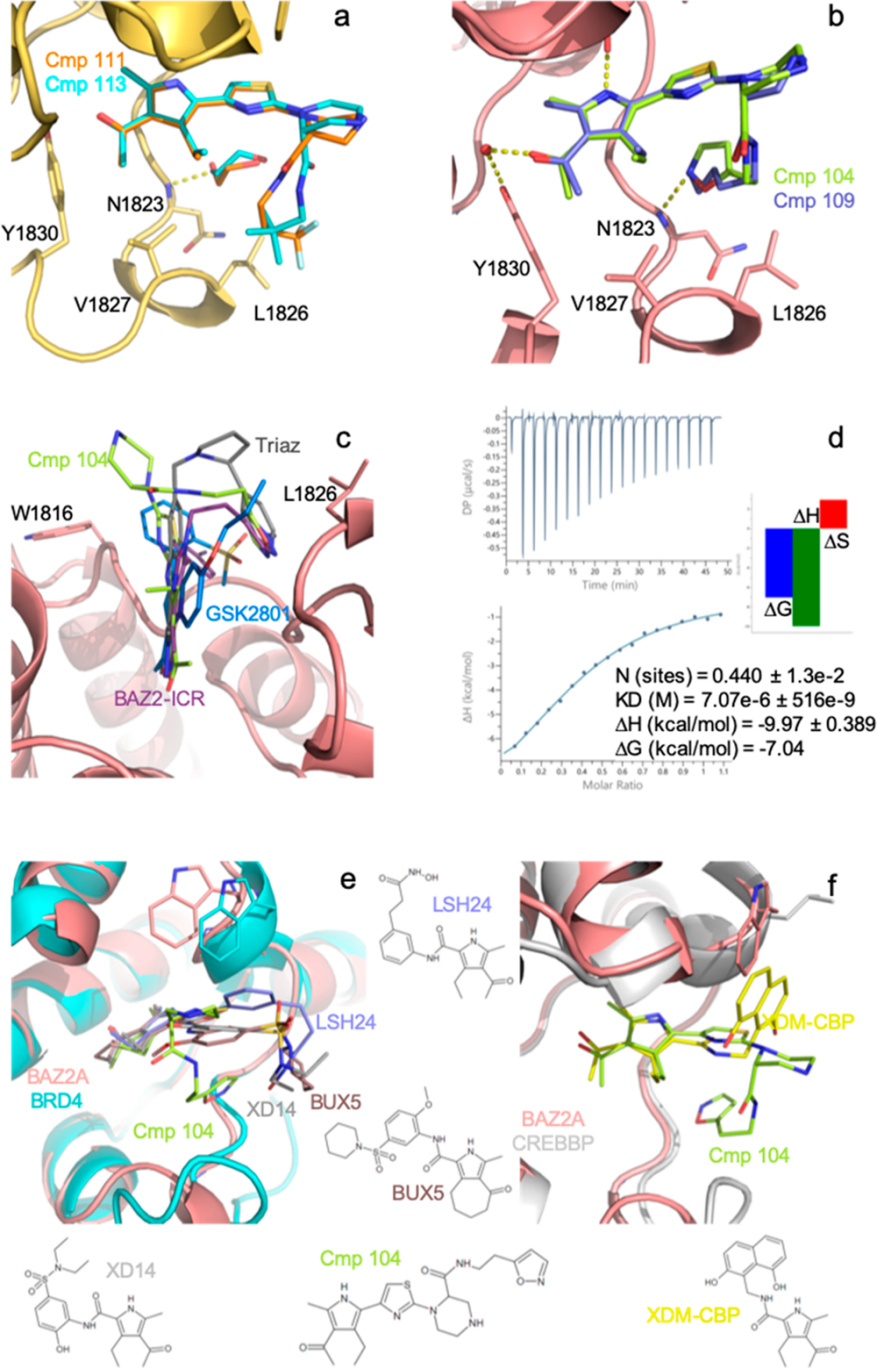
(a) Tails of compounds **111** and **113** provide additional contacts with Leu1826 and Val1827. (b) Compounds **104** and **109** form a hydrogen bond with the Asn1823 main chain. (c) An intramolecular stacking is observed in **104**, as similarly obtained with BAZ2-ICR (PDB 7BL8) and the triazole compound **21** of ref (16). (d) ITC titration for compound **104** showing an enthalpically driven binding restricted to a single enantiomer. (e, f) Compound **104**’s tail is oriented differently with respect to those of previously developed acetylpyrrole inhibitors directed against BRD4 (e) and CREBBP (f).

The thermodynamic parameters for compound **104** binding to the BAZ2A bromodomain were finally characterized by isothermal titration calorimetry (ITC). ITC confirmed the observed enantioselectivity showing that the active fraction is about half of the racemic mixture (Figure 5d). The equilibrium dissociation constant (*K*_D_) of 7.1 μM is in good agreement with the IC_50_ of 4.0 μM measured by AlphaScreen. The presence of 0.25% DMSO in the ITC experiment leads to a slightly less favorable *K*_D_ value as DMSO competes for binding to the conserved Asn.^18,19^ Binding is dominated by a relevant enthalpic contribution partially counteracted by an entropic penalty in accordance with the various interactions observed in the crystal structure combined with the freezing of the ethyl-isoxazole tail. Finally, **104** shows good selectivity over the closely related BAZ2B bromodomain, and the highest achieved so far, with an IC_50_ of 33.5 μM, as determined by AlphaScreen (Figure S6). As compared with previous screening campaigns,^8,11,17^ such selectivity was obtained in the context of a reasonable LE and optimal log *P*, log *D*, and LLE.

The acetylpyrrole headgroup has already been explored in BRD4 and CREBBP inhibitors (Figure 5e,f), also cross-reacting with BRD7, BRD9, and HDACs.^20−22^ However, compound **104** features a unique thiazole ring as scaffold. Furthermore, its binding mode is different, as its ethyl-isoxazole tail folds back in the bromodomain Kac pocket and stacks with the thiazole ring.

The extensive chemical space exploration for the derivatization of the acetylpyrrole-thiazole scaffold toward a BAZ2A hit compound allows drawing various conclusions.

First, the pay-off for the inhibitors’ extension toward the binding pocket rim or outside it in the region of Trp1816 or Glu1820 is limited, at least in terms of potency. Nonetheless, positively charged groups are better accepted and provide selectivity over the paralog BAZ2B bromodomain. A more significant gain in potency can be instead obtained moving toward the more hydrophobic region of the pocket rim in the area of Leu1826, Val1827, and Phe1872. The best reward is finally obtained by saturating the pocket, with a substantial profit deriving from both additional interactions with the protein matrix (i.e., Asn1823) and intramolecular aromatic stacking in the inhibitor, generating the four-layer sandwich with Trp1816 and Leu1826.

As observed for other bromodomains,^23−28^ the BAZ2A pocket can be targeted enantiospecifically. This, other than providing a selectivity determinant, also offers excellent negative controls for further characterization of the BAZ2A mechanism of action. In conclusion, the hit compound **104** has an IC_50_ of 4 μM (AlphaScreen) and a *K*_D_ of 7 μM (ITC). The enthalpic contribution to the binding free energy of compound **104** is nearly −10 kcal/mol, which originates from its favorable interactions with the bromodomain and the stacking of its isoxazole and thiazole rings. Furthermore, we have disclosed a set of 23 holo crystal structures and related binding affinity values for compounds that bear a positively charged functional group. These data can be used to benchmark docking algorithms and scoring functions for the correct treatment of ionic groups partially or fully exposed to solvent (Supporting Information).

## Supporting Information Available

Tables S1 and S2, 2D structures of compounds **1**–**73**; Tables S3–S6, X-ray data collection and refinement statistics for all determined crystallographic structures; Figure S1, structural alignment; Figures S2–S6, AlphaScreen single-dose tests and dose–response curves for selected compounds; and Figure S7, *F*_o_ – *F*_c_ electron density maps for compounds whose structure in complex with the BAZ2A bromodomain are described herein (PDF)

## Accession Codes

BAZ2A structures were deposited to the PDB with accession numbers 7QZT (**9**), 7QVT (**13**), 7R01 (**18**), 7QVU (**25**), 7QVV (**36**), 7QWU (**44**), 7QWF (**45**), 7R0B (**47**), 7QWY (**61**), 7QX2 (**63**), 7QX9 (**65**), 7QXL (**77**), 7QYE (**78**), 7QYO (**79**), 7QYT (**80**), 7QZ0 (**83**), 7QZ4 (**87**), 7QYU (**88**), 7QZB (**98**), 7QZC (**104**), 7QYV (**109**), 7QYW (**111**), and 7QZI (**113**). Atomic coordinates and experimental data will be released upon article publication.

## Abbreviations

BAZ2A: bromodomain adjacent to zinc finger domain protein 2A
BET: bromodomain and extra-terminal domain
EZH2: enhancer of zeste homolog 2
Kac: acetylated lysine
NoRC: nucleolar remodeling complex
TNBC: triple-negative breast cancer
