# Supplementary material for: Identification of a BAZ2A-Bromodomain Hit Compound by Fragment Growing
Source: ACS Med Chem Lett. 2022 Aug 3;13(9):1434–43. doi: 10.1021/acsmedchemlett.2c00173 (PMC9465710; doi:10.1021/acsmedchemlett.2c00173)
Supplement: Supplementary file 1 — ml2c00173_si_001.pdf [file ml2c00173_si_001.pdf]

Identification of a BAZ2A-bromodomain hit compound by fragment growing

Andrea Dalle Vedove[1], Giulia Cazzanelli[1], Laurent Batiste[2], Jean-Rémy Marchand[2], Dimitrios Spiliotopoulos[2§], Jessica Corsi[1], Vito Giuseppe D'Agostino[1], Amedeo Caflisch[2*] and Graziano Lolli[1*]

Author Affiliations:

[1]Department of Cellular, Computational and Integrative Biology - CIBIO, University of Trento, via Sommarive 9, 38123 Povo - Trento, Italy

[2]Department of Biochemistry, University of Zürich, Winterthurerstrasse 190, CH-8057 Zürich, Switzerland

[§]Current address: Xenometrix AG, Gewerbestrasse 25, CH-4123, Allscwhil, Switzerland

[*]*Correspondence to Amedeo Caflisch and Graziano Lolli*: caflisch@bioc.uzh.ch; graziano.lolli@unitn.it.

Table of Contents

Experimental Procedures..S2

Table S1 – Chemical structures of selected fragments...S4

Table S2 – Chemical structures of selected small molecules...................................S6

Tables S3-S6 – Data Collection and Refinement Statistics………………..……………..S8

Figure S1 – Structural alignment……………………..S10

Figures S2-S6 – Inhibitory activity by AlphaScreen…...S11

Figure S7 – F_o-F_c OMIT maps…..……………………..S16

References……………….…..……………………...S17

EXPERIMENTAL PROCEDURES

Chemicals

All compounds were purchased from either Enamine Ltd. or SIA Chemspace with a purity > 95%.

Virtual library preparation and Molecular Docking

Compared to previous fragment screening campaigns for BAZ2A [8, 16], we extended the size of the library by including fragments with up to three rotatable bonds. Including molecules with up to 3 rotors provides additional diversity in the limited chemical space of rigid fragments. We further filtered the library with compounds bearing a basic center, with the intent of targeting Glu1820 as a potential selectivity lever between BAZ2A vs other bromodomains.

The docking was carried out with the program SEED [14-15, 29]. Briefly, the compounds were parametrized using CGenFF [30-31] and up to 100 conformers for each compound were generated with the ETKDG algorithm of the RDKit [32]. The threshold of 100 conformers per molecule is very generous for a library with a maximum of three rotors per molecule, and generates enough conformer diversity in order to contain the most likely metastable states in water for these rather rigid molecules. The BAZ2A structure (PDB code 5MGJ with Glu1820 in the pt rotameric configuration) was kept rigid during docking. The presence of a hydrogen bond between the ligand docking pose and the Asn1873 side chain was used as the only constraint for the search space, which was not limited to any particular cavity definition. This allowed scanning the pocket rim for favorable interactions. As in a previous docking campaign [11], the multiple poses of the compounds were ranked according to the electrostatic interaction energy with an approximation of solvent screening effects by the generalized Born model. For the latter, the Born radii were calculated by a numerical procedure [33].

Protein purification, X-ray Crystallography and competition binding assay

BAZ2A bromodomain, either wild-type or the E1845H/L1848S crystallization-prone mutant, was produced as previously reported [8].

Trigonal crystals were obtained by co-crystallization as described in the same work. Monoclinic crystals were obtained by co-crystallization with compound **4** of reference [17]; a back-soaking procedure was then applied by incubating the crystal in the crystallization solution without inhibitors for 36-48 hours, and subsequently exposing it to the new ligand of interest for 12-24 hours. Diffraction data were collected at the Elettra Synchrotron Light Source (Trieste, Italy), XRD1 and XRD2 beamlines, and at the ESRF (Grenoble, France), ID30-A1 beamline. Data were processed and structures were solved as described elsewhere [34]. BAZ2A/cmp**9** data have been corrected for diffraction anisotropy with STARANISO (https://staraniso.globalphasing.org/cgi-bin/staraniso.cgi). Data collection and refinement statistics are reported in Suppl. Tables 3-6. Electron densities ($2F_o$-F_c polder OMIT map) for the bound inhibitors are shown in Suppl. Fig. 7.

AlphaScreen assay was conducted as detailed in [11]. All experiments were performed at least in duplicate.

Isothermal Titration Calorimetry

ITC experiments were performed using an MicroCal PEAQ-ITC microcalorimeter (Malvern Panalytical, UK) at 25°C with a stirring speed of 750 rpm in 20mM Tris pH 7.8, 0.2 M NaCl, 0.2 mM TCEP and 0.25% DMSO. For each experiment, one 0.4 µl injection followed by eighteen 2 µl injections have been performed, with 150 seconds separating each injection. TN40-25 was tested at 70 µM in the cell with His-Baz2A in the syringe at 400 µM. A blank run with only the buffer was carried out. Data analysis was done with PEAQ-ITC Analysis software, fitting the curve with the 'one site' model.

Table S1. Selected fragments from the molecular docking campaign.

compound	structure	compound	structure	compound	structure
**1**	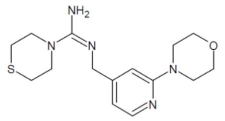	**13**	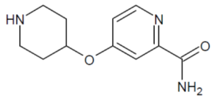	**25**	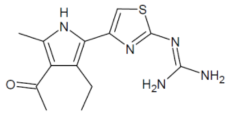
**2**	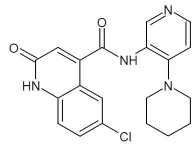	**14**	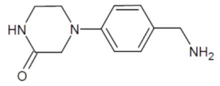	**26**	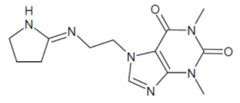
**3**	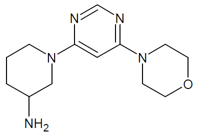	**15**	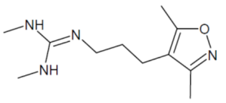	**27**	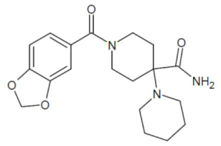
**4**	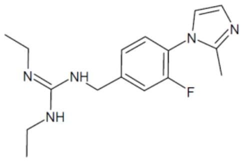	**16**	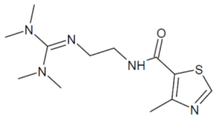	**28**	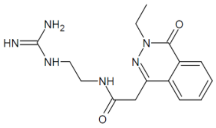
**5**	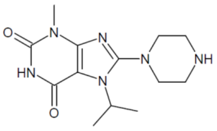	**17**	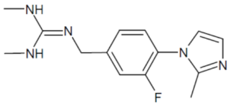	**29**	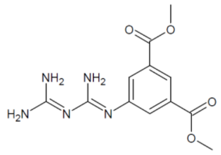
**6**	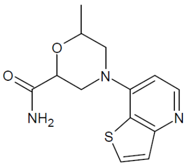	**18**	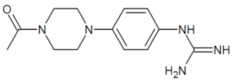	**30**	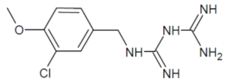
**7**	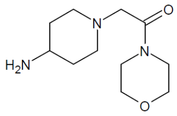	**19**	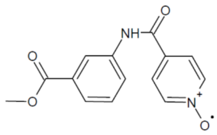	**31**	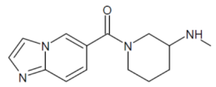
**8**	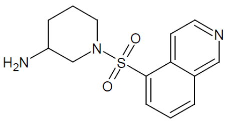	**20**	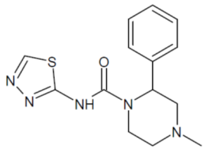	**32**	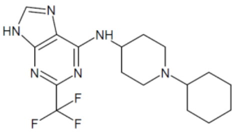
**9**	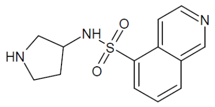	**21**	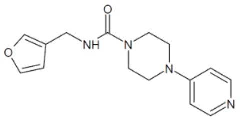	**33**	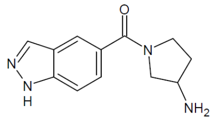
**10**	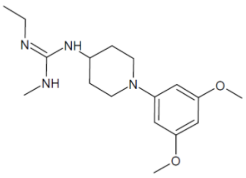	**22**	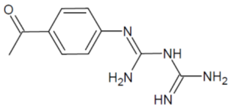	**34**	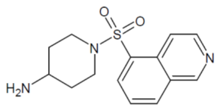
**11**	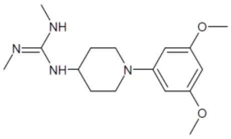	**23**	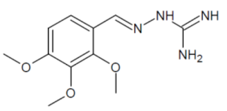	**35**	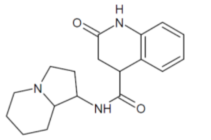

**12**	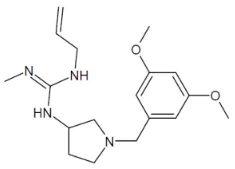	**24**	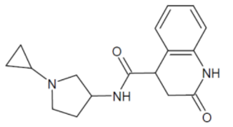	**36**	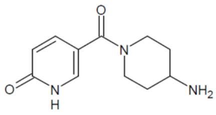

Table S2. Selected fragments for the optimization campaign.

compound	structure	compound	structure	compound	structure
**37**	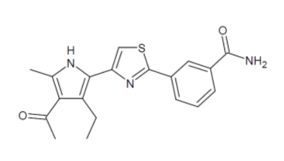	**50**	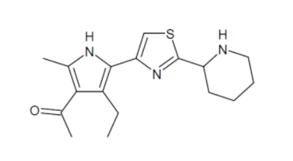	**63**	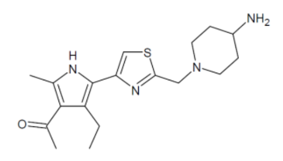
**38**	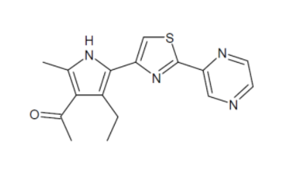	**51**	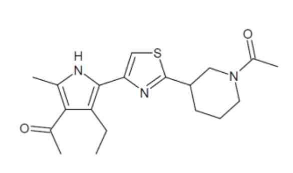	**64**	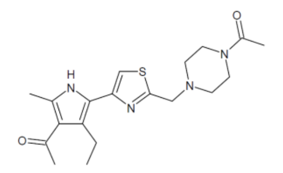
**39**	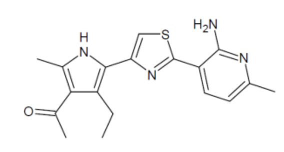	**52**	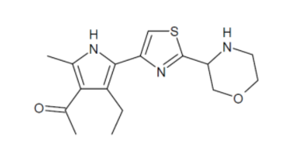	**65**	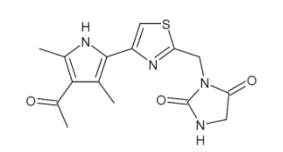
**40**	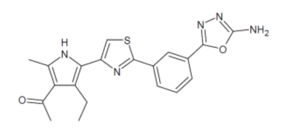	**53**	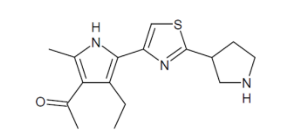	**66**	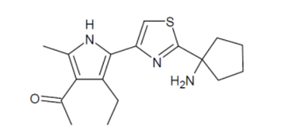
**41**	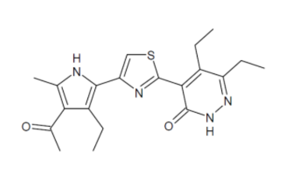	**54**	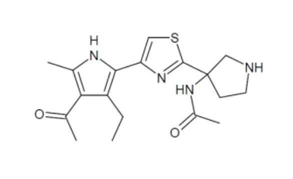	**67**	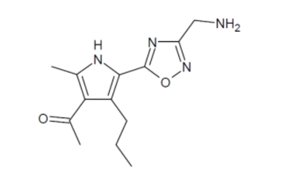
**42**	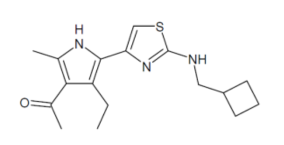	**55**	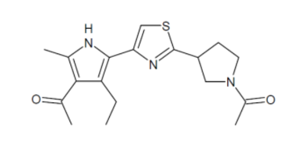	**68**	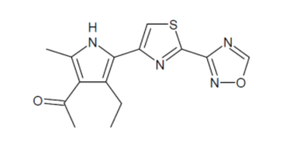
**43**	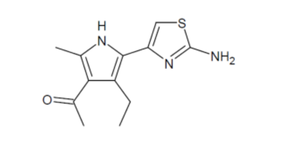	**56**	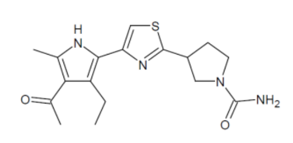	**69**	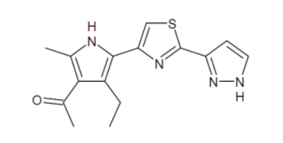
**44**	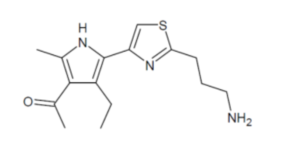	**57**	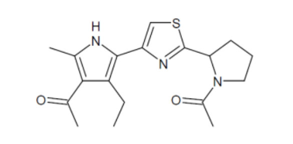	**70**	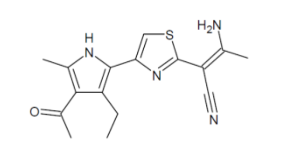
**45**	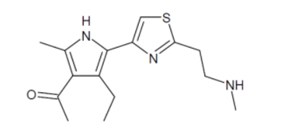	**58**	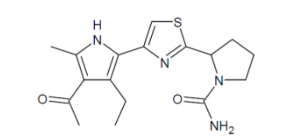	**71**	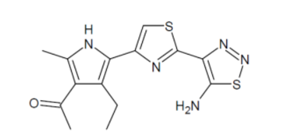
**46**	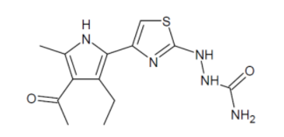	**59**	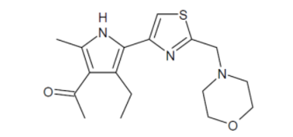	**72**	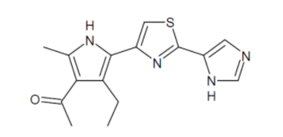
**47**	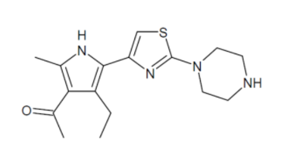	**60**	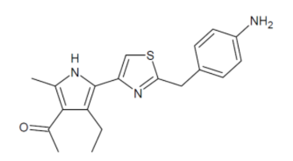	**73**	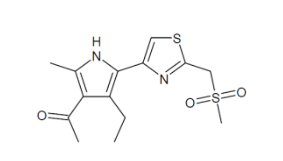

**48**	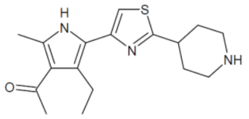	**61**	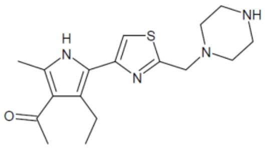		
**49**	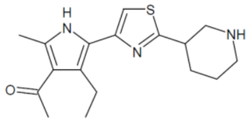	**62**	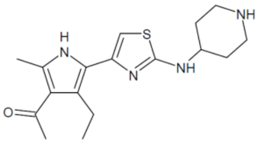		

Table S3. Data Collection and Refinement Statistics for BAZ2A structures in complex with fragments

	Cmp **9**	Cmp **13**	Cmp **18**	Cmp **25**	Cmp **36**
Data Collection					
Beamline	ESRF ID30-A1	Elettra XRD1	ESRF ID30-A1	Elettra XRD1	Elettra XRD1
Space group	$P3_121$	$P3_121$	$P3_121$	$P3_121$	$P3_121$
Unit-cell parameters (Å, °)	a = 94.76 b = 94.76 c = 32.74	a = 95.14 b = 95.14 c = 32.72	a = 94.65 b = 94.65 c = 32.74	a = 95.77 b = 95.77 c = 32.13	a = 91.45 b = 91.45 c = 32.63
Wavelength (Å)	0.97	1.00	0.97	1.00	1.00
Resolution (Å)	82.06-2.18 (2.48-2.18)	47.57-2.60 (2.72-2.60)	81.97-2.26 (2.33-2.26)	47.89-2.40 (2.49-2.40)	45.72-2.60 (2.72-2.60)
R_{merge} (%)	6.2 (32.2)	25.6 (170.4)	4.2 (30.9)	30.0 (157.9)	20.9 (140.8)
R_{meas} (%)	8.1 (41.4)	26.9 (179.1)	5.0 (36.8)	31.6 (165.4)	22.1 (148.6)
R_{pim} (%)	5.2 (25.8)	8.2 (54.7)	2.6 (19.5)	9.6 (49.0)	6.9 (47.1)
$\langle I/\sigma(I) \rangle$	12.2 (3.8)	8.8 (1.7)	15.6 (3.2)	7.5 (1.7)	10.6 (2.0)
$CC^{1/2}$	0.998 (0.909)	0.992 (0.747)	0.999 (0.916)	0.993 (0.774)	0.994 (0.661)
Sph. complet. (%)	60.1 (19.1)	100.0 (100.0)	93.9 (98.4)	100.0 (100.0)	99.9 (100.0)
Ell. complet. (%)	86.0 (75.8)				
Multiplicity	3.0 (2.8)	10.6 (10.5)	3.2 (3.3)	10.8 (11.2)	10.1 (9.8)
Refinement					
Resolution (Å)	41.04-2.18	41.20-2.60	47.34-2.26	41.48-2.40	30.17-2.60
R_{work}/R_{free} (%)	18.3/23.5	20.8/24.7	17.9/21.0	19.9-24.1	22.6/26.1
R.m.s. deviations					
Bond lengths (Å)	0.009	0.004	0.006	0.007	0.006
Bond angles (°)	0.97	0.60	0.81	0.90	1.09
PDB entry	7QZT	7QVT	7R01	7QVU	7QVV

Table S4. Data Collection and Refinement Statistics for BAZ2A structures in complex with acetylpyrrole fragments

	Cmp **44**	Cmp **45**	Cmp **47**	Cmp **61**	Cmp **63**	Cmp **65**
Data Collection						
Beamline	Elettra XRD2	Elettra XRD1	Elettra XRD1	Elettra XRD2	Elettra XRD2	Elettra XRD2
Space group	$P2_1$	$P3_121$	$P3_121$	$P3_121$	$P2_1$	$P2_1$
Unit-cell parameters (Å, °)	a = 37.52 b = 34.90 c = 37.56 β = 92.14	a = 93.88 b = 93.88 c = 33.03	a = 94.23 b = 94.23 c = 33.26	a = 94.36 b = 94.36 c = 33.06	a = 37.27 b = 35.07 c = 37.90 β = 93.35	a = 37.53 b = 34.96 c = 37.56 β = 91.88
Wavelength (Å)	0.94	1.00	0.97	0.91	1.00	1.00
Resolution (Å)	37.54-1.60 (1.63-1.60)	46.94-2.25 (2.32-2.25)	47.11-2.35 (2.43-2.35)	47.18-2.44 (2.54-2.44)	37.84-1.43 (1.45-1.43)	37.54-1.50 (1.53-1.50)
R_{merge} (%)	6.3 (67.4)	14.6 (127.9)	13.8 (133.1)	15.6 (137.6)	7.4 (19.8)	8.6 (71.7)
R_{meas} (%)	6.9 (73.6)	15.4 (133.9)	14.2 (137.2)	16.1 (141.8)	8.1 (21.9)	9.4 (78.1)
R_{pim} (%)	2.7 (29.1)	4.7 (39.4)	3.3 (33.2)	3.7 (33.8)	3.2 (9.2)	3.7 (30.7)
$\langle I/\sigma(I) \rangle$	17.9 (2.6)	13.0 (2.4)	17.0 (2.5)	15.6 (2.7)	15.2 (6.0)	9.8 (2.0)
$CC^{1/2}$	0.999 (0.838)	0.998 (0.861)	0.999 (0.867)	0.999 (0.948)	0.996 (0.985)	0.996 (0.822)
Completeness (%)	99.9 (99.1)	100.0 (100.0)	100.0 (100.0)	99.9 (99.7)	99.2 (93.7)	99.8 (100.0)
Multiplicity	6.3 (6.2)	10.6 (11.3)	18.8 (16.8)	19.0 (17.1)	6.2 (5.3)	6.2 (6.3)
Refinement						
Resolution (Å)	37.51-1.60	40.66-2.25	47.11-2.35	47.18-2.44	37.84-1.43	37.54-1.50
R_{work}/R_{free} (%)	19.5/23.7	19.9/24.2	17.1/20.8	21.8/25.4	13.4/15.6	18.3/21.4
R.m.s. deviations						
Bond lengths (Å)	0.006	0.007	0.006	0.008	0.008	0.009
Bond angles (°)	0.92	0.90	0.84	1.05	1.10	1.04
PDB entry	7QWU	7QWF	7R0B	7QWY	7QX2	7QX9

Table S5. Data Collection and Refinement Statistics for BAZ2A structures in complex with acetylpyrrole derivatives

	Cmp **77**	Cmp **78**	Cmp **79**	Cmp **80**	Cmp **83**	Cmp **87**
Data Collection						
Beamline	Elettra XRD2	Elettra XRD2	Elettra XRD2	Elettra XRD2	Elettra XRD2	Elettra XRD2
Space group	$P2_1$	$P2_1$	$P2_1$	$P3_121$	$P3_121$	$P3_121$
Unit-cell parameters (Å, °)	a = 37.46<br>b = 34.96<br>c = 37.83<br>β = 92.74	a = 37.46<br>b = 35.02<br>c = 37.65<br>β = 92.47	a = 37.39<br>b = 35.07<br>c = 37.91<br>β = 93.05	a = 94.00<br>b = 94.00<br>c = 33.08	a = 94.63<br>b = 94.63<br>c = 33.01	a = 94.28<br>b = 94.28<br>c = 33.09
Wavelength (Å)	1.00	0.94	0.91	0.94	1.00	1.00
Resolution (Å)	37.78-1.15 (1.17-1.15)	37.61-1.65 (1.68-1.65)	37.86-0.98 (1.00-0.98)	47.00-2.60 (2.72-2.60)	81.95-2.10 (2.16-2.10)	81.65-2.30 (2.38-2.30)
R_{merge} (%)	5.8 (51.1)	11.8 (45.7)	3.3 (34.4)	19.8 (192.3)	11.0 (154.6)	20.7 (155.5)
R_{meas} (%)	6.4 (57.9)	12.8 (49.9)	3.6 (38.6)	20.3 (197.3)	11.3 (159.6)	21.3 (161.6)
R_{pim} (%)	2.6 (26.5)	5.0 (19.7)	1.5 (17.3)	4.6 (43.8)	2.6 (38.9)	5.0 (43.3)
$<I/\sigma(I)>$	14.5 (3.1)	8.3 (3.2)	25.1 (3.6)	16.5 (1.9)	21.9 (2.1)	10.4 (1.8)
$CC^{1/2}$	0.998 (0.910)	0.993 (0.957)	0.999 (0.949)	0.999 (0.791)	0.999 (0.696)	0.995 (0.764)
Completeness (%)	99.5 (95.9)	99.8 (100.0)	99.4 (97.5)	100.0 (100.0)	99.4 (99.7)	100.0 (99.9)
Multiplicity	5.9 (4.6)	6.2 (6.3)	6.0 (4.8)	19.3 (20.1)	18.9 (16.2)	18.1 (13.5)
Refinement						
Resolution (Å)	37.78-1.15	37.61-1.65	27.33-0.98	47.00-2.60	47.33-2.10	47.15-2.30
R_{work}/R_{free} (%)	16.0/18.0	18.1/20.9	13.0/13.6	21.9/25.7	17.4/20.4	18.4/22.1
R.m.s. deviations						
Bond lengths (Å)	0.007	0.006	0.008	0.003	0.005	0.007
Bond angles (°)	1.04	1.05	1.35	0.76	0.80	0.94
PDB entry	7QXL	7QYE	7QYO	7QYT	7QZ0	7QZ4

Table S6. Data Collection and Refinement Statistics for BAZ2A structures in complex with acetylpyrrole derivatives

	Cmp **88**	Cmp **98**	Cmp **104**	Cmp **109**	Cmp **111**	Cmp **113**
Data Collection						
Beamline	Elettra XRD2	Elettra XRD2	Elettra XRD2	Elettra XRD2	Elettra XRD2	Elettra XRD2
Space group	$P2_1$	$P3_121$	$P3_121$	$P3_121$	$P3_121$	$P3_121$
Unit-cell parameters (Å, °)	a = 37.13<br>b = 35.47<br>c = 37.86<br>β = 92.49	a = 94.20<br>b = 94.20<br>c = 33.09	a = 94.13<br>b = 94.13<br>c = 33.23	a = 93.90<br>b = 93.90<br>c = 33.23	a = 94.56<br>b = 94.56<br>c = 33.17	a = 94.20<br>b = 94.20<br>c = 33.21
Wavelength (Å)	0.94	1.00	1.00	1.00	0.97	1.00
Resolution (Å)	37.83-1.50 (1.53-1.50)	81.58-2.15 (2.21-2.15)	81.52-2.10 (2.16-2.10)	81.32-2.25 (2.32-2.25)	81.89-2.10 (2.16-2.10)	81.58-1.98 (2.03-1.98)
R_{merge} (%)	6.6 (73.4)	17.7 (148.9)	16.7 (213.5)	13.1 (137.5)	9.4 (125.4)	11.5 (192.6)
R_{meas} (%)	7.3 (80.4)	18.3 (154.5)	17.2 (219.4)	13.5 (142.5)	9.7 (131.1)	11.8 (198.2)
R_{pim} (%)	2.9 (32.4)	4.4 (40.6)	3.9 (50.6)	3.1 (36.6)	2.3 (37.4)	2.7 (46.7)
$<I/\sigma(I)>$	15.1 (2.2)	13.5 (2.1)	14.3 (1.8)	18.9 (2.6)	19.4 (2.1)	19.5 (2.1)
$CC^{1/2}$	0.999 (0.894)	0.998 (0.617)	0.999 (0.768)	0.999 (0.865)	1.000 (0.864)	0.999 (0.831)
Completeness (%)	99.2 (99.0)	99.7 (96.6)	100.0 (100.0)	100.0 (99.9)	100.0 (100.0)	99.9 (100.0)
Multiplicity	6.2 (6.0)	16.7 (14.0)	19.3 (18.6)	18.4 (15.2)	17.9 (12.1)	19.1 (17.9)
Refinement						
Resolution (Å)	27.08-1.50	47.11-2.15	47.08-2.10	30.77-2.25	47.29-2.10	47.11-1.98
R_{work}/R_{free} (%)	15.9/20.3	17.6/21.0	18.0/20.8	19.2/23.7	18.1/20.9	17.7/20.3
R.m.s. deviations						
Bond lengths (Å)	0.008	0.006	0.005	0.004	0.005	0.006
Bond angles (°)	1.04	0.87	0.84	0.80	0.82	0.86
PDB entry	7QYU	7QZB	7QZC	7QYV	7QYW	7QZI

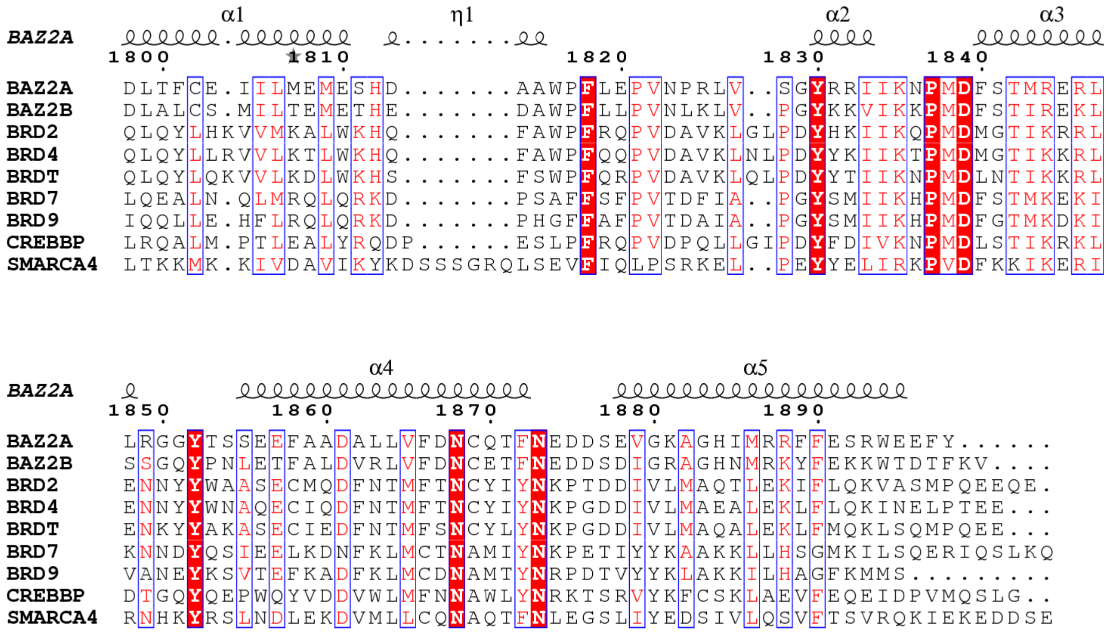

Figure S1. Structural alignment of representative bromodomains. Glu1820 was selected as possible selectivity determinant for BAZ2A. Alignment was realized with ESPript. Robert, X.; Gouet, P. Deciphering key features in protein structures with the new ENDscript server. *Nucleic Acids Res.* **2014**, 42, W320-324.

.

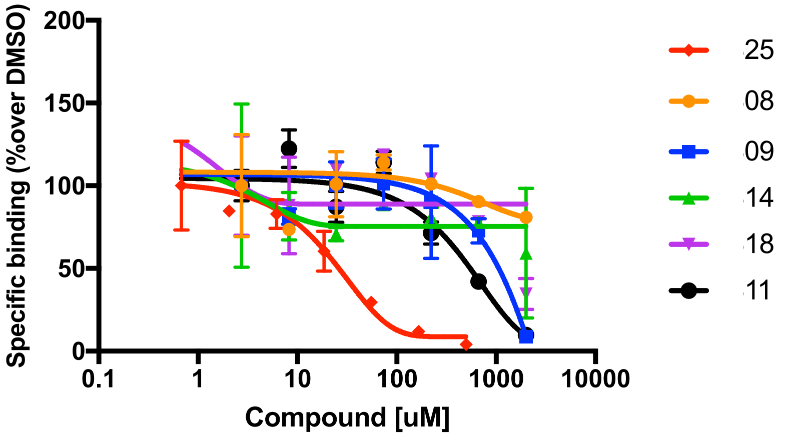

Figure S2. AlphaScreen competition binding assay for initially selected fragments. The specific binding to the acetylated peptide relative to the control DMSO (y-axis) is plotted against the corresponding compound concentration in μM in log10 scale (x-axis).

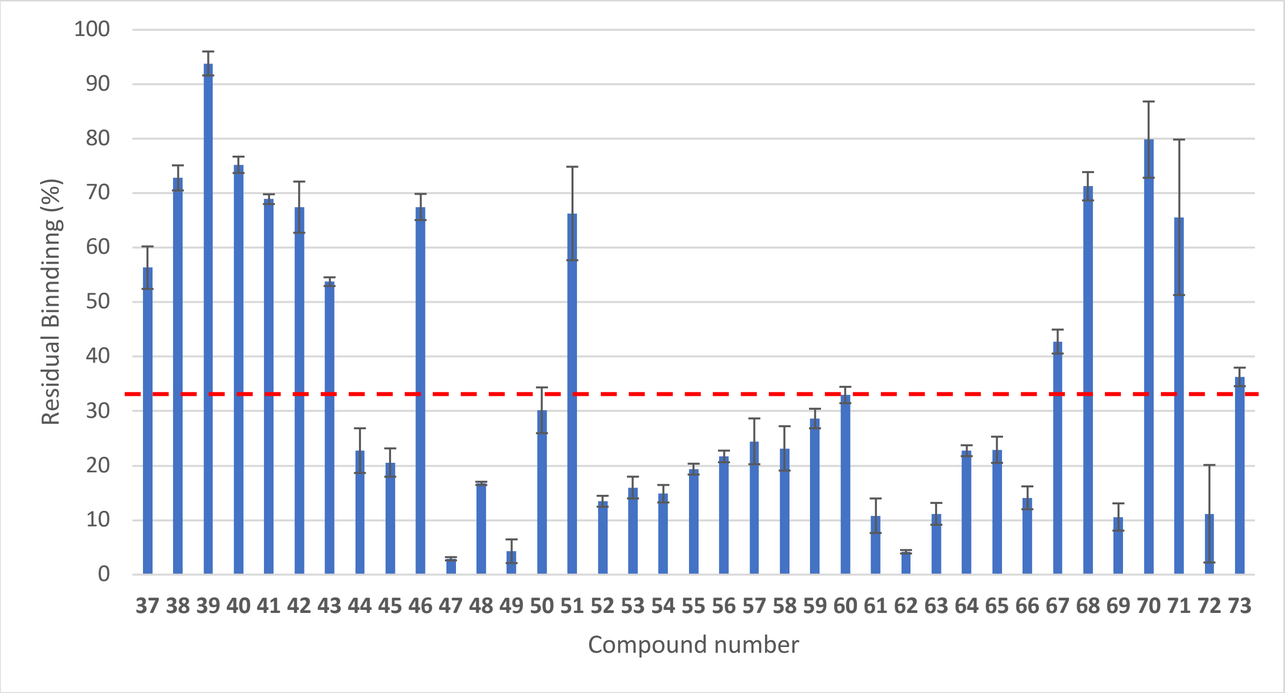

Figure S3. Single-dose (500 µM) AlphaScreen competition binding assay for fragments selected for the optimization campaign. Residual binding is reported in percentage.

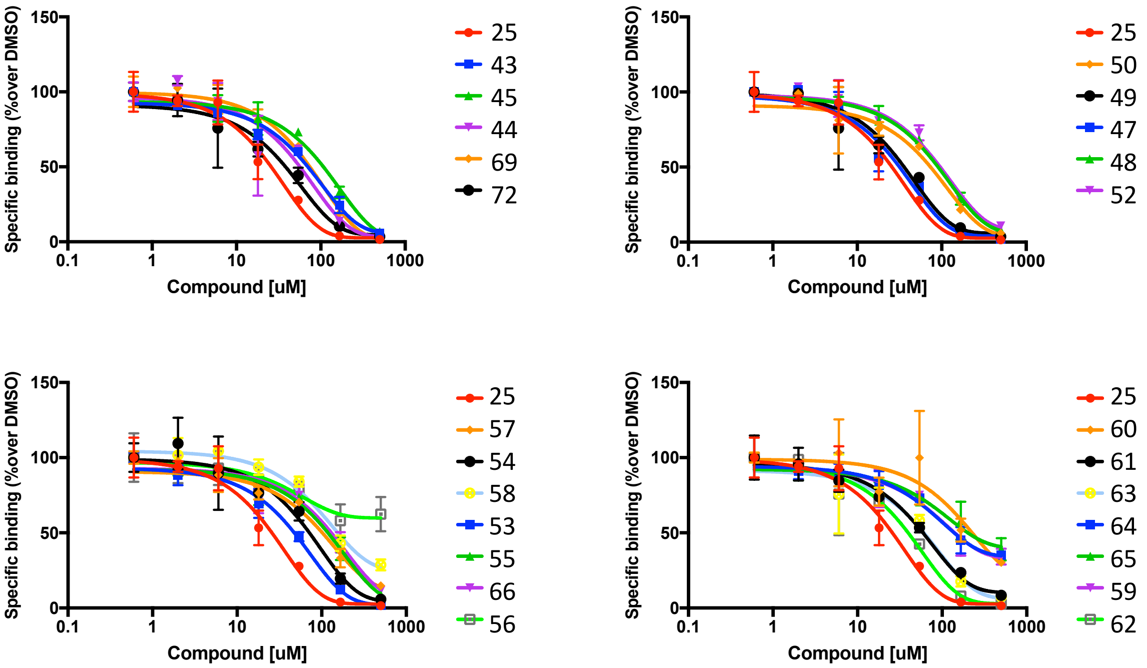

Figure S4. Dose-response AlphaScreen competition binding assay for fragments selected for the optimization campaign. Residual binding is reported in percentage. The specific binding to the acetylated peptide relative to the control DMSO (y-axis) is plotted against the corresponding compound concentration in μM in log10 scale (x-axis).

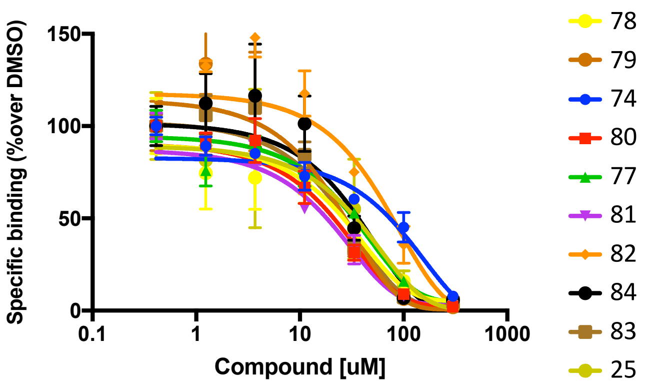

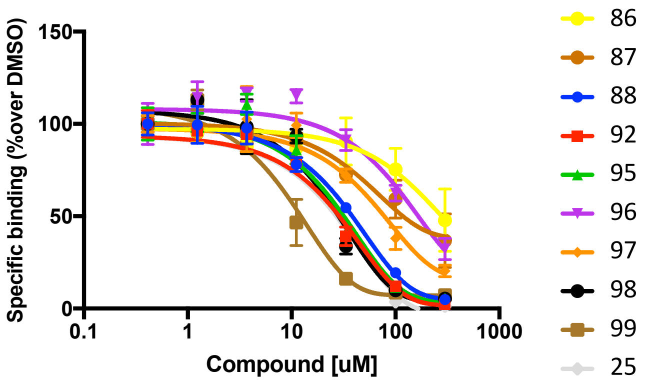

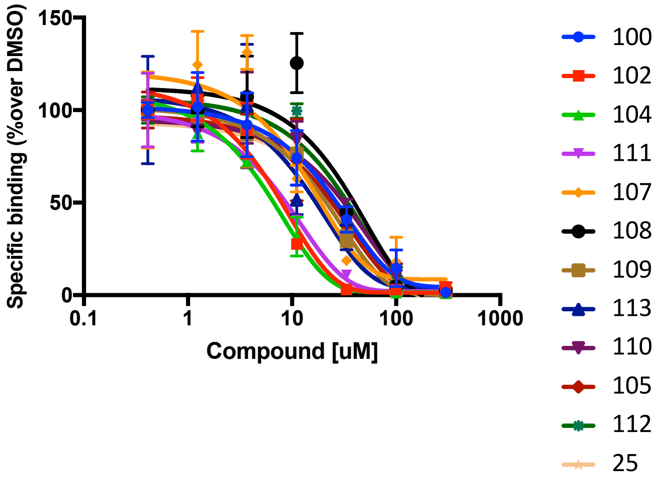

Figure S5. Dose-response AlphaScreen competition binding assay for molecules selected for the fragment growing campaign. Residual binding is reported in percentage. The specific binding to the acetylated peptide relative to the control DMSO (y-axis) is plotted against the corresponding compound concentration in µM in log10 scale (x-axis).

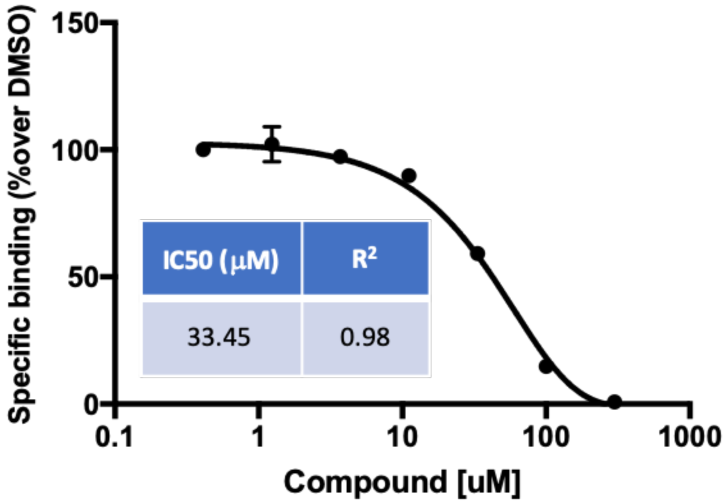

Figure S6. Dose-response AlphaScreen competition binding assay for compound **104** on the BAZ2B bromodomain. Residual binding is reported in percentage. The specific binding to the acetylated peptide relative to the control DMSO (y-axis) is plotted against the corresponding compound concentration in µM in log10 scale (x-axis).

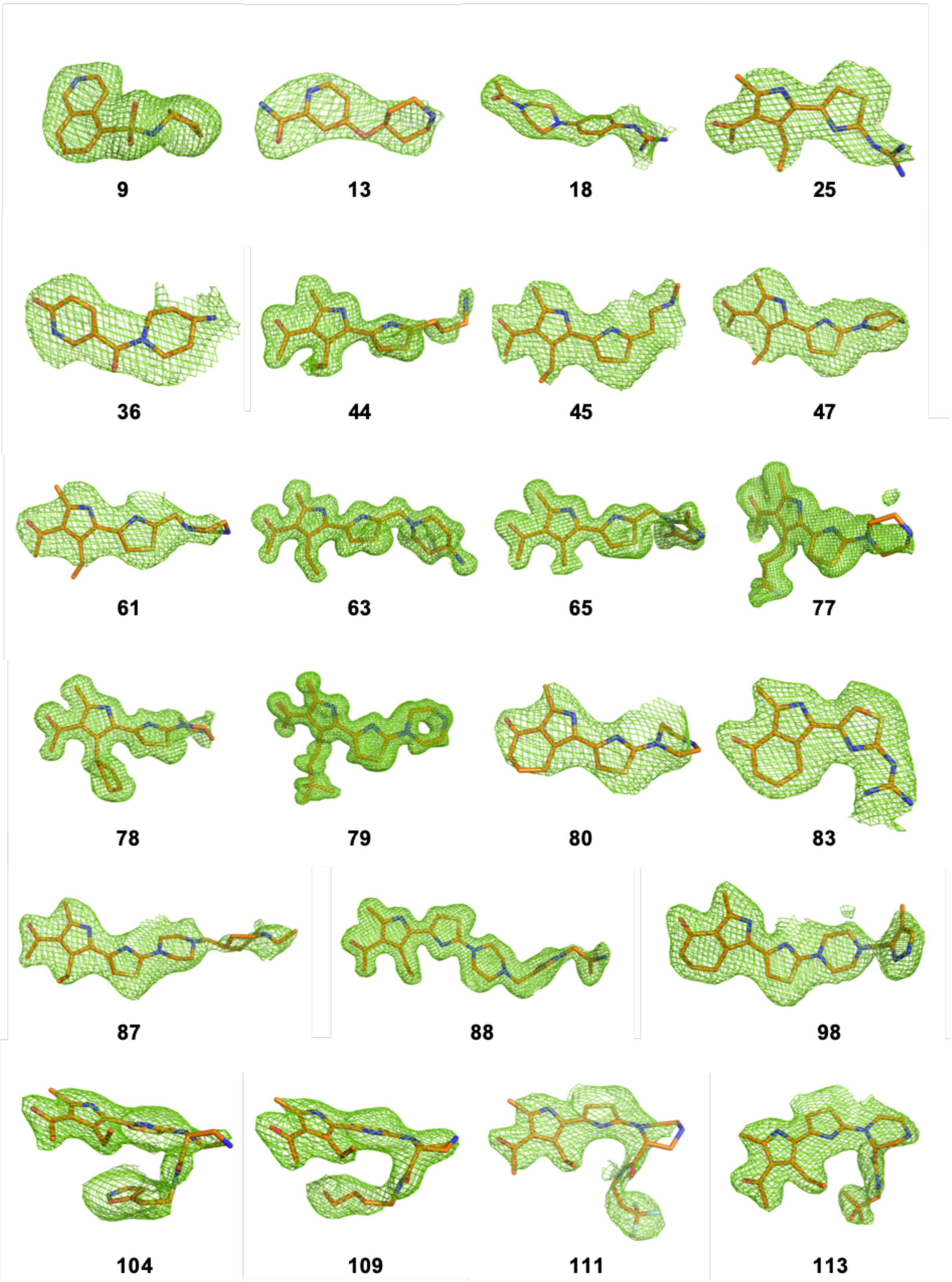

Figure S7. Electron densities for the reported compounds. F_o-F_c polder OMIT maps (green) are contoured at 2.3 to 3.0 σ. Liebschner, D.; Afonine, P. V.; Moriarty, N. W.; Poon, B. K.; Sobolev, O. V.; Terwilliger, T. C.; Adams, P. D. Polder maps: improving OMIT maps by excluding bulk solvent. *Acta Crystallogr. D Struct. Biol.* **2017**, *73*, 148-157.

REFERENCES

29. Cecchini, M.; Kolb, P.; Majeux, N.; Caflisch, A. Automated docking of highly flexible ligands by genetic algorithms: a critical assessment. *J. Comput. Chem.* **2004**, *25*, 412-422.

30. Vanommeslaeghe, K.; MacKerell, A. D. Jr. Automation of the CHARMM General Force Field (CGenFF) I: Bond Perception and Atom Typing. *J. Chem. Inf. Model.* **2012**, *52*, 3144−3154.

31. Vanommeslaeghe, K.; Raman, E. P.; MacKerell, A. D. Jr. Automation of the CHARMM General Force Field (CGenFF) II: Assignment of Bonded Parameters and Partial Atomic Charges. *J. Chem. Inf. Model.* **2012**, *52*, 3155−3168.

32. Landrum, G. RDKit: Open-source cheminformatics. rdkit.org

33. Scarsi, M.; Apostolakis, J.; Caflisch, A. Continuum electrostatic energies of macromolecules in aqueous solutions. *J. Phys. Chem. A* **1997**, ***101***, 8098-8106.

34. Lolli, G.; Caflisch, A. High-Throughput Fragment Docking into the BAZ2B Bromodomain: Efficient in Silico Screening for X-Ray Crystallography. *ACS Chem. Biol.* **2016**, *11*, 800-807.
